# Global Trends of Latent Prostate Cancer in Autopsy Studies

**DOI:** 10.3390/cancers13020359

**Published:** 2021-01-19

**Authors:** Takahiro Kimura, Shun Sato, Hiroyuki Takahashi, Shin Egawa

**Affiliations:** 1 Department of Urology, The Jikei University School of Medicine, Tokyo 105-8461, Japan; s-egpro@jikei.ac.jp; 2 Department of Pathology, The Jikei University School of Medicine, Tokyo 105-8461, Japan; casinodrive@jikei.ac.jp (S.S.); hawk1bridge@gmail.com (H.T.)

**Keywords:** latent cancer, prostate cancer, autopsy

## Abstract

**Simple Summary:**

The incidence of prostate cancer (PC) is statistically biased due to the increase in prostate-specific antigen (PSA) screening and the accuracy of national cancer registration systems. However, studies on latent PC provide less biased information. This comprehensive review included studies evaluating latent PC in several countries. The prevalence of latent PC has been stable since 1950 in Western countries, but it has increased over time in Asian countries. Latent PC in Asian men has increased in prevalence and is higher in grade. This increase occurred not only due to the increase in PSA screening, but also due to increasing adoption of a Westernized lifestyle. Racial differences between Caucasian and Asian men may also explain the tumor location of latent PC. The autopsy findings in patients with latent PC included a significant proportion of high grade and stage cancers, suggesting a need to reconsider the definition of clinically insignificant PC.

**Abstract:**

The incidence of prostate cancer (PC) has been increasing in Asian countries, where it was previously low. Although the adoption of a Westernized lifestyle is a possible explanation, the incidence is statistically biased due to the increase in prostate-specific antigen (PSA) screening and the accuracy of national cancer registration systems. Studies on latent PC provide less biased information. This review included studies evaluating latent PC in several countries after excluding studies using random or single-section evaluations and those that did not mention section thickness. The findings showed that latent PC prevalence has been stable since 1950 in Western countries, but has increased over time in Asian countries. Latent PC in Asian men has increased in both prevalence and number of high-grade cases. Racial differences between Caucasian and Asian men may explain the tumor location of latent PC. In conclusion, the recent increase in latent PC in Asian men is consistent with an increase in clinical PC. Evidence suggests that this increase is caused not only by the increase in PSA screening, but also by the adoption of a more Westernized lifestyle. Autopsy findings suggest the need to reconsider the definition of clinically insignificant PC.

## 1. Introduction

The incidence of prostate cancer (PC) has been increasing globally in recent years. It is the second most frequently diagnosed cancer and the fifth leading cause of cancer-related deaths among men worldwide [1]. The incidence of PC in recent decades has been heavily influenced by the emergence of prostate-specific antigen (PSA) testing. The availability of PSA testing from the middle to the late 1980s led to the intensive use of the test for screening, with a subsequent rapid increase in the incidence rate in Western countries. This trend has also been growing in Asian countries, where the incidence of PC was previously low [1,2]. The cause of this increase in Asian countries is thought to be multifactorial. Although the spread of PSA screening may be a major cause, changes in lifestyle due to more Westernized diets might be another [3,4]. The accuracy of national cancer registration systems may also influence the incidence, as national cancer registration has not been developed in some Asian countries. However, PC mortality has been decreasing in many Western countries, possibly linked to earlier diagnosis due to PSA screening and improved treatment. In contrast, PC mortality is increasing in several Asian and developing countries [1]. These reports may support the influence of changes in risk factors due to more Westernized lifestyles in such countries.

Studies on latent PC provide less biased information about PC incidence compared to studies on clinical PC. Latent PC is defined as PC that is first detected in autopsy without any clinical signs of PC during the patient’s lifetime. Since Mintz and Smith first reported latent PC in 13% of 100 autopsied cases in 1934 [5], many studies have been reported globally. A recent meta-analysis of 29 studies from 1948 to 2013 indicated that while the prevalence of latent PC significantly increases with age, there is no obvious time trend [6]. However, the time trend of latent PC prevalence might differ among countries. For example, a more recent study indicated that the prevalence of latent PC in Japanese men has been increasing [7]. In addition, a prospective study comparing latent PC in Asian and Caucasian men indicated that the prevalence in Asian men did not differ significantly from that in Caucasian men [8]. These results suggest that not only recent efforts for early detection, such as PSA screening, but also the change to Westernized diets and lifestyles may have influenced the increase in PC in Asian countries.

Information from studies on latent PC provides important insights from a different viewpoint. This review comprehensively discusses the results of latent PC studies in Western and Asian countries.

## 2. Potential Biases in Methodology in Latent PC Studies

As the methodology for latent PC studies has not yet been standardized and there are several biases between the studies, careful evaluation of their methods is required for precise interpretation. First, study populations, subject sources, and inclusion criteria differed among the studies. While most of the studies involved autopsies performed in hospitals, other studies assessed forensic autopsies. In addition, some studies have analyzed databases of institutional autopsy records or national or regional autopsy registries. However, a meta-analysis indicated that the source of subjects (population vs. hospital-based) was not significantly associated with the prevalence of latent PC [6]. Age was significantly associated with latent PC prevalence, which increased with each decade of age [6]. Thus, the inclusion and exclusion criteria of age and its distribution significantly influenced the prevalence of latent PC. Race is another major factor that affects the prevalence of latent PC. These results must be presented separately in studies that include various races. The methods of sample preparation also differed among the studies. The time elapsed from death to autopsy, step-sectioning versus random and/or single-section evaluation, and the interval between sections in step sectioning can also influence the prevalence of latent PC. The prevalence was reportedly higher in step-sectioned tissues than in randomly divided tissues [9], whereas there was no evidence that the section thickness or delay of autopsy affect PC prevalence [6]. However, information regarding the delay of autopsies is limited in most studies. The methods of diagnosis such as central review or not and use of immunohistochemical evaluation may also differ among studies, although a meta-analysis concluded that the use of immunohistochemistry was not associated with PC prevalence [6].

## 3. Prevalence of Latent PC in Western Countries

The most important topic in autopsy studies was the prevalence of latent PC. After the first report by Mintz and Smith in 1934, many such studies have been conducted in Western countries [5]. Studies evaluating latent PC by step-sectioning of the prostate in the US and Europe are listed in Table 1 [8,10,11,12,13,14,15,16,17,18,19,20,21,22,23,24,25,26,27,28,29,30,31,32,33]. Studies using random or single-section evaluations and those that did not mention section thickness were excluded. Cohorts of different nationalities or races are listed separately even if they were reported within the same study. The prevalence of latent PC varied from 9.6% to 58.6% between the studies. The ranges and distributions of age also varied between the studies. For example, some studies included men under 20 years of age [25,29], while another study included only men older than 70 years [15]. Age influenced the prevalence of latent PC, as it was one of the most significant factors associated with prevalence [6,34]. A recent meta-analysis of 29 studies reported an estimated mean cancer prevalence at age < 30 years of 5% (95% confidence interval (CI): 3–8%), which increased nonlinearly to 59% (95% CI: 48–71%) by age > 79 years [6]. Race is another factor that affects prevalence. Six studies in the US reported the prevalence of latent PC in Caucasian and Black men separately [15,22,23,25,26,33], with reported prevalence rates of 25.9–58.6% and 19.4–43.3%, respectively. All four studies that conducted statistical analyses on the prevalence of latent PC in Caucasian and Black men concluded that racial differences did not exist. However, these results require careful interpretation, as age distributions may differ between these races, especially in forensic studies. A recent review of 19 studies including 6,024 autopsies suggested a racial difference in latent PC prevalence between Caucasian and Black men (35.7% vs. 50.5%), but did not conduct a statistical analysis [34].

**Figure 1:**
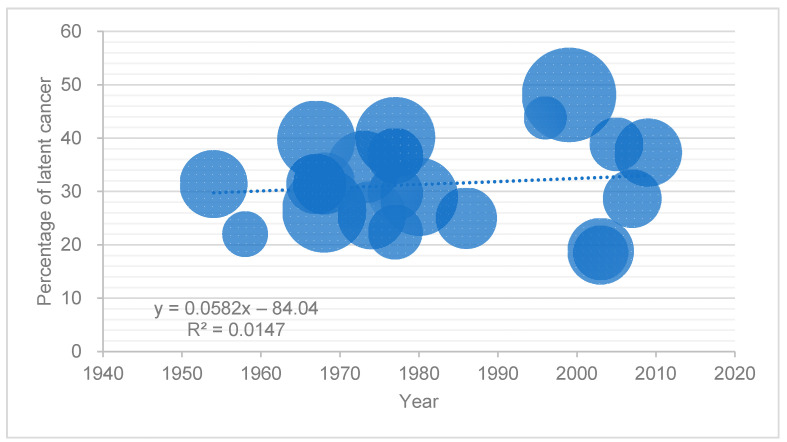
Prevalence of latent PC in studies of Caucasians in the US and Europe.

Figure 1 shows the prevalence of latent PC in studies of Caucasians in the US and Europe published after 1950 by year of publication. The size of each point was proportional to the number of men included in each study. The analytic linear approximation line of the datapoints indicated that the latent PC prevalence was stable over time. The spread of PSA screening programs is thought to have increased the diagnosis of insignificant PC and decreased the prevalence of latent PC. However, few studies have examined the changes in the prevalence of latent PC before and after the PSA era. A retrospective study using an autopsy record database from a single institution in the US reported that the prevalence of latent PC decreased three-fold with the widespread use of PSA screening [35]. In this study, the prevalence was 4.8% in men older than 40 years between 1955 and 1960, compared to 1.2% between 1991 and 2001. However, this study was limited by the lack of whole-mount sections to examine the prostate, which might lead to a lower prevalence compared to those in other autopsy studies using step-sectioning. However, the prevalence of latent PC in Japan has increased despite the spread of PSA screening, although the exposure rate of PSA testing in Asian countries is still lower than that in Western countries [2,7]. The trends in Asian countries are discussed in Section 4.

The prevalence of latent PC in studies of US and European Caucasians published after 1950 by year of publication. The size of each point was proportional to the number of men included in each study.

## 4. The prevalence of Latent PC in Asian and Other Countries

Studies investigating latent PC are fewer in Asian countries than in Western countries. In 1937, Yotsuyanagi et al. first reported a 3% prevalence of latent PC in Japanese men in a domestic journal [36]. Among the literature published in international journals, in 1961, Karube first reported a latent PC prevalence of 10.9% in Japanese men older than 40 years by step-sectioning [36]. Studies on latent PC in Asia and Africa are listed in Table 2 [7,8,19,20,23,36,37,38,39,40,41,42,43,44]. Studies using random or single-section evaluations that were not published in English were excluded. Some results are part of a multinational study. Most studies in Asia were from Japan [7,8,19,23,36,38,39,40], with the exception of two studies from Singapore [20,37] and one each from China [41], Hong Kong [20], and Iran [42]. Reports from other regions include Jamaica in Latin America and Uganda in Africa as part of a multinational study in 1977 [20]. The prevalence of latent PC in Jamaica and Uganda was 32.7% and 24.0%, respectively, which were higher than those in Asian countries in the same reports (15.0% and 14.5% in Hong Kong and Singapore, respectively). In addition, the mean age of men in Uganda was 58.3 years, which was 5 years younger than of those in other countries (64.5 in Singapore, 63.4 in Hong Kong, and 63.2 in Jamaica). Updated data for prevalence in Latin America and Africa are required.

**Figure 2:**
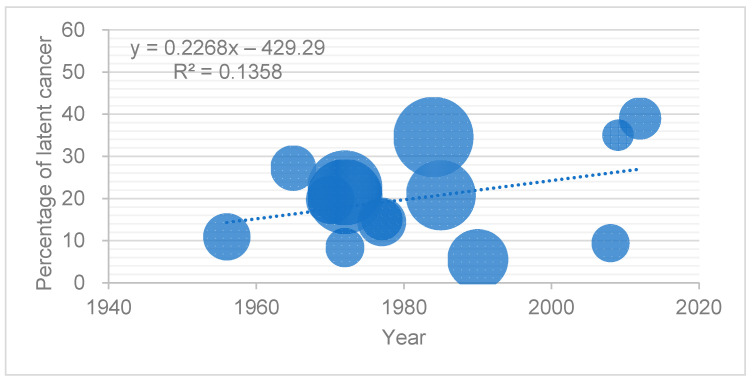
Prevalence of latent PC in the studies on Asian populations. Prevalence of latent PC in studies of Asian countries published after 1950 by year of publication. The size of each point was proportional to the number of men in the study.

Figure 2 shows the prevalence of latent PC in studies of Asian countries published after 1950. Data from the study conducted by Takahashi et al. in Japan were excluded because they focused on men older than 90 years of age [40]. The size of each point is proportional to the number of men in the study. The analytic linear approximation line of the datapoints indicated that the prevalence of latent PC in Asia increased over time compared to that in the US and Europe (Figure 1). The prevalence was 8.3–27.2% from the 1960s to 1970s, 5.5–34.6% from the 1980s to the 1990s, and 9.4–39.0% after 2000. Two Japanese studies directly compared the time trends in latent PC within the same institutions. Yatani et al. compared the latent PC prevalence within the same institution between men from 1965 to 1979 and from 1982 to 1986, both in pre-PSA era periods. The prevalence increased significantly from 22.5% to 34.6% [39]. More recently, Kimura et al. compared the prevalence of latent PC between Japanese men in pre- and post-PSA eras. The prevalence in men was 20.8% in 1983–1987 and 43.3% in 2008–2013 [7]. Both studies indicated a significant increase in higher-grade and larger cancers. Yatani et al. reported a higher rate of infiltrative tumors in the cohort in 1965–1979 than in 1982–1986, at 9.8% and 17.8%, respectively [39]. Kimura et al. reported a significantly larger index cancer volume in men in 2008–2013 compared to that in 1983–1987 [7].

The increased prevalence of latent PC in Asian men is consistent with the increased prevalence of clinical PC [45]. A major explanation for the increase in latent PC in Asian countries may be lifestyle changes due to more Westernized diets. The incidence of clinical PC in US men of Japanese ancestry in 1973–1986 was between that of Caucasians in the US and Japanese men born in Japan within the same period, suggesting the influence of both genetic and lifestyle factors on PC incidence [46]. In contrast, a comparative study published in 1973 showed that the age-adjusted prevalence of latent PC did not differ significantly between Japanese men in Japan and those in Hawaii (20.5% and 26.7%, respectively). However, the age-adjusted prevalence of the proliferative type of latent PC was higher in Japanese Hawaiians than in native Japanese (19.1% and 8.7%, respectively) [19].

Zlotta et al. prospectively compared the prevalence of latent PC in 100 Japanese men and 220 Russian men [8]. The prevalence was 35.0% and 37.3% in Japanese and Russian men, respectively, and did not differ significantly. However, Japanese men had a greater probability of having a PC Gleason score (GS) of 7 than Russian men after adjusting for age and prostate weight. These results suggest the increasing prevalence and grade of latent PC in Asian men over the past few decades.

## 5. Pathological Findings from Latent PC

Although latent PC does not cause clinical symptoms and is not generally detected during the lifetime, most studies showed that a significant proportion of latent PC had high-grade, capsular, or seminal vesicle invasion. In studies in the US and Europe, 5.43% of cancers were GS 7 or greater and 11–13% were pT3 or greater [8,27,29,30,31]. In contrast, in Asian studies, 35.7–51.4% of latent PC was GS 7 or higher, with proportions higher than those in Western reports, although the proportion of cancers with pT3 or greater was similar (11.5–12.7%) [7,8,42]. Consequently, these cases of latent PC included clinically significant cancer as defined by Epstein (the presence of T3 or greater and/or index tumor volume of 500 mm^3^ or greater and/or GS ≥ 7 [47]). A prospective comparative study by Zlotta et al. reported that 29.3% and 51.4% of latent PC cases were clinically significant in Russian and Japanese men, respectively [8]. A comparative study of contemporary latent PC and historical controls in Japan reported an index cancer volume of 500 mm^3^ in 9.6% of cancers in men in 1983–1987 and 25.5% in 2008–2013, a significant difference [7]. The increase in proportion of significant cancer in latent PC notwithstanding the spread of PSA screening might suggest an increase in high-grade cancer in Asian countries, especially in Japan. These results also suggest the need to reexamine the definition of clinically insignificant PC. Stamey et al. defined clinically significant PC as organ-confined tumors of <0.5 cm^3^, GS 3+3 with no grade 4 or 5 [48]. However, it can also be defined as a cancer that does not affect the patient during the natural course of his lifetime. The requirements of Stamey’s definition may be too stringent.

Investigating the tumor location of latent PC could improve our understanding of the origin of PC and how it grows [49]. Racial differences between Caucasian and Asian men have been suggested to affect not only the prevalence, but also the tumor location of PC. A comparative study of radical prostatectomy specimens reported that 35.5% and 0.6% of PCs originated in the transition zone (TZ) in Japanese men and US men, respectively [50]. Studies that categorized tumor location into anterior or posterior regions reported that anterior cancer was more prevalent in Asian men than in Caucasian men [50,51,52,53,54,55,56]. However, most studies evaluating tumor location in the prostate have analyzed only prostatectomy specimens. The tumor location of the prostatectomy specimens may overestimate the prevalence in the peripheral zone (PZ) or posterior cancer because of its higher detectability by digital rectal examination and transrectal prostate biopsy compared to in the TZ or anterior cancer. In this sense, the tumor location in latent PC may be less biased. There are limited reports regarding tumor location in latent PC. An autopsy study in Hungary including 139 men aged 18–95 years reported a latent PC prevalence of 38.8%; among the 64 tumor foci, 82.8% and 18.9% were present in the PZ and TZ, respectively [29]. Another study in the US including 164 men aged 54–73 years reported that latent PC was present in 29% of the cases, with 62% and 36% of PCs located in the posterior and anterior regions, respectively, and 77% and 16%—in the PZ and central zone in the prostate, respectively [31].

Reports on tumor locations of latent PC in Asian men are limited. A report of 149 autopsies of Iranian men over 50 years of age detected invasive adenocarcinoma in 14 (9.4%) cases, including nine cases (64%) in the posterior region, one case (7%) in the anterior region, and four cases (29%) in both lobes of the prostate [42]. A report of 182 Japanese men observed latent PC in 39.0% of cases, occurring in the TZ, PZ, or without dominance in 38.0%, 57.8%, and 4.2% of cases, respectively [43]. The tumors were located in the anterior and posterior regions in 49.3% and 40.8% of the cases, respectively. Approximately 40% of the tumors were located in the TZ and anterior region of the prostate, a rate higher than that reported in Western studies. The age distribution also differed between TZ and PZ cancers. In elderly men, cancer is more frequently diagnosed in the PZ than in the TZ [43]. This was consistent with the report by Takahashi et al. on autopsies in men over 90 years of age, which revealed that all latent PCs were localized in the PZ of the prostate [40]. An autopsy study in the US reported that most TZ cancers showed a different pathological pattern from that of PZ cancers, with lower GS and less aggressiveness [57]. However, a Japanese study reported that the pathological features did not differ between the TZ and PZ and between anterior and posterior cancers in terms of GS, tumor volume, or prevalence of clinically significant cancer; however, there is variation in the pT stage—PZ cancer has a significantly higher pT stage than TZ cancer. Several anatomical explanations have been proposed to explain this difference. For example, the TZ is separated from the surrounding area by fibromuscular tissue, whereas no such structure exists in the PZ. Moreover, the TZ contacts with the prostate capsule from the outside to the back of the PZ, and T3b cases of the TZ are few because of the anatomical position [43]. However, a prospective comparative study of Japanese and Russian autopsy cases reported similar tumor locations between cohorts, in which latent PC was located in the TZ in 25.9% and 20.7% and in the anterior region in 20.0% and 21.9% of the cases in Japanese and Russian men, respectively. Further investigation is required to determine whether there is a racial difference in tumor location and whether the location in Asian men has changed due to Western diet and lifestyle.

Few studies have investigated tumor location in the vertical direction. In their international multicenter study investigating 1327 autopsies from seven counties or regions, Breslow et al. reported latent PC in 350 cases. In the vertical direction, more tumors were present at the middle and apex levels than at the base. However, the evaluation method to describe the tumor distribution has not yet been standardized, and further studies are warranted.

Most latent PC cases represent the less aggressive forms of PC. Thus, comparing molecular markers or genomic aberrations between latent and clinical PCs is an ideal method to investigate their effects. Igawa et al. reported a significantly higher nm23-HI gene expression level in clinical PC than in normal prostatic tissues, latent PC, and clinical PC [58]. Watanabe et al. reported that *Ras* gene mutations in latent PC varied among ethnic groups and that the frequency in Japanese men was higher than that in US Black or Caucasian men [59]. Alipov et al. compared the expression of the ETS1 proto-oncogene in latent PC, benign prostatic hyperplasia, normal prostatic tissues, and clinical PC [60], reporting negative expression in benign tissues and higher levels in clinical PC than in latent PC. Maekawa et al. investigated the TMPRSS2 Met160Val polymorphism in Japanese men, including 518 men with sporadic PC, 433 healthy controls, and 154 men with latent PC [61]. The TMPRSS2 Met160Val polymorphism is a genetic risk factor for sporadic PC but not for latent PC in the Japanese population. However, molecular studies using latent PC are limited, possibly because of the limited quality and availability of latent PC specimens.

## 6. Limitations of Autopsy Studies

Latent PC has a unique cancer status compared to the malignancies of other origins. Although it has been investigated for a long time, several problems remain to be solved. Section 5 described variations in the methodologies used for sample preparation and diagnosis. Although step-sectioning of the whole prostate is a standard method, the duration from death to autopsy is difficult to control. A central review for diagnosis is mandatory because of inter- and intra-observer variability for the pathological diagnosis of PC [62]. A fundamental bias also exists in autopsy studies. As the subjects were men who died in the hospital, their backgrounds differed from those of healthy men. Inaba et al. reported that 106 of the 182 autopsy cases (58.2%) had been performed due to death of malignancy other than PC (unpublished data).

The available data on latent PC were provided by a limited number of countries and regions. Most studies were from North America, Western Europe, and Japan, whereas data from Africa and Latin America are limited. More importantly, the number of autopsies has been steadily declining over the past 30–40 years worldwide [63]. After the 2000s, the autopsy rate was only 7–9% in the US, compared to approximately 25–35% in the mid-1960s and 50% of all hospital deaths in the 1940s and 1950s [64,65]. In Japan, more than 40,000 autopsies were performed in 1985, but this number had gradually decreased to approximately 10,000 by 2018 [66].

One explanation for the limited number of studies evaluating molecular markers in latent PC is the low quality of specimens from autopsies. RNA and proteins were extracted during the time between death and autopsy. To overcome such limitations in autopsy studies, rapid autopsies have emerged [67]. In this new methodology, tissues are collected as soon as possible after the patient’s death. Ideally, the quality of a rapid autopsy tissue can be considered comparable to the quality of a fresh surgical biopsy tissue.

## 7. Learning from Latent PC and Future Directions

While latent PC studies have a long history, the available evidence remains limited. Latent PC studies have revealed a larger prevalence of insignificant PC than the incidence of clinical PC. PC prevalence increases with age and more than half of both Caucasian and Asian men over 80 years of age have indolent PC. The recent increase in latent PC in Asian men is consistent with an increase in clinical PC in Asian countries. These findings suggest that this increase in clinical PC in Asian countries is due not only to the spread of PSA screening, but also to the adoption of Westernized lifestyles.

In addition, the results of autopsy studies suggest the need to reconsider the definition of clinically insignificant PC, which is thought to be an ideal candidate for active surveillance. The present definition might be too strict, as latent PC included a significant proportion of cancer cases thought to be life-threatening, such as with GS ≥ 7 and pT3 or greater. Cancer volume and the percentage of high-grade cancer cases also increased with age. However, the individuals lived without the influence of PC throughout their lives. Molecular analyses are required in latent PC studies to distinguish between indolent and life-threatening PC. Methodologies such as rapid autopsies have opened the door for new studies of latent PC.

## Figures and Tables

**Table 1:** Studies evaluating latent PC by step-sectioning of prostate in the US and Europe.

Author	Year Published	Country/Ethnicity	Duration of Study	Study Population	No. of Cases	Age	No. of Cancers	%	Pathology Section Width (mm)	Ref. No.
Moore	1935	Austria	1931–1932	Hospital	304	Range, 21–90	52	16.7	4	[10]
Andrews	1949	UK	NA	Hospital	142	Range, 40–79	17	12.0	4	[11]
Edwards	1953	Canada	1942–1945	Hospital	173	Mean, 64.1	35	16.7	4	[12]
Franks	1954	US	NA	Forensic	220	NA	69	31.4	4	[13]
Viitanen	1958	Finland	NA	Hospital	100	≥50	22	22.0	5	[14]
Halpert	1965	US/Black	NA	Hospital	30	Range, 70–79	13	43.3	4	[15]
Halpert	1965	US/Caucasian	NA	Hospital	70	Range, 70–79	41	58.6	4	[15]
Liavag	1968	Norway	NA	Hospital	340	≥40	90	26.5	4	[16]
Lundberg	1970	Sweden	1967	Hospital	292	NA	116	39.7	5	[17]
Harbitz	1973	Norway	1967–1968	Hospital	172	≥40	54	31.4	4–6	[18]
Akazaki	1973	US/men of Japanese ancestry	1969–1972	Hospital	158	≥50	46	29.1	3	[19]
Breslow	1977	Germany	NA	Hospital	145	Mean, 65	43	29.7	5	[20]
Breslow	1977	Israel	NA	Hospital	143	Mean, 65	32	22.4	5	[20]
Breslow	1977	Sweden	NA	Hospital	306	Mean, 65	123	40.2	5	[20]
Hølund	1980	Denmark	1971–1977	Hospital	223	Range, 36–94	57	25.6	3	[21]
Gulleyardo	1980	US/Black	NA	Hospital	207	NA	65	31.4	3	[22]
Gulleyardo	1980	US/Caucasian	NA	Hospital	293	NA	85	29	3	[22]
Yatani	1982	Colombia	1967–1970	Hospital	182	Mean, 64.4	NA	31.5	3	[23]
Yatani	1982	US/Black	1969–1978	Hospital	178	Mean, 63.6	NA	36.9	3	[23]
Yatani	1982	US/Caucasian	1969–1978	Hospital	253	Mean, 63.2	NA	34.6	3	[23]
Yatani	1982	US/men of Japanese ancestry	1969–1978	Hospital	417	Mean, 70.1	NA	25.6	3	[23]
Stemmermann	1992	US/men of Japanese ancestry	1970–1990	Hospital	293	Mean, 67.9	80	27.3	3	[24]
Sakr	1993	US/Black	NA	Forensic	98	10–50	19	19.4	3–4	[25]
Sakr	1993	US/Caucasian	NA	Forensic	54	10–50	14	25.9	3–4	[25]
Brawn	1996	US/Black	NA	Hospital	15	≥50	5	33.3	3	[26]
Brawn	1996	US/Caucasian	NA	Hospital	89	≥50	39	43.8	3	[26]
Sanchez-Chapado	2003	Spain	NA	Forensic	146	Mean, 48.5	27	18.5	3-4	[28]
Soos	2005	Hungary	NA	Hospital	139	18–95	54	38.8	4	[29]
Stamtiou	2007	Greece	2002–2004	Hospital	212	≥30	40	18.8	4	[30]
Haas	2007	US (92% Caucasian)	NA	Hospital	164	Median, 64	47	28.7	4	[31]
Polat	2009	Turkey	NA	Hospital	114	Mean, 55	11	9.6	4	[32]
Powell	2010	US/Black	1993–2004	Forensic	630	20–79	NA	35.1	2.5	[33]
Powell	2010	US/Caucasian	1993–2004	Forensic	426	20–79	NA	48.1	2.5	[33]
Zlotta	2013	Russia	2008–2011	Hospital	220	Mean, 62.5	82	37.3	4	[8]

NA: not available.

**Table 2:** Studies evaluating latent PC by step-sectioning of prostate in Asian and other countries.

Author	Year Published	Country/Ethnicity	Duration of Study	Study Population	No. of Cases	Age	No. of Cancers	%	Pathology Section Width (mm)	Ref. No.
Karube	1961	Japan	1954–1959	Hospital	229	≥40	25	10.9	4–5	[36]
Lee	1972	Singapore	NA	Hospital	156	Range, 42–87	13	8.3	4	[37]
Akazaki	1973	Japan	1969–1972	Hospital	239	≥50	47	19.7	3	[19]
Bean	1973	Japan	1961–1969	Hospital	213	≥50	58	27.2	5	[38]
Breslow	1977	Hong Kong	NA	Hospital	173	Mean, 65	26	15.0	5	[20]
Breslow	1977	Jamaica	NA	Hospital	168	Mean, 65	55	32.7	5	[20]
Breslow	1977	Singapore	NA	Hospital	242	Mean, 65	35	14.5	5	[20]
Breslow	1977	Uganda	NA	Hospital	150	Mean, 65	36	24.0	5	[20]
Yatani	1982	Japan	1965–1979	Hospital	576	Mean, 67.7	NA	20.5	3	[23]
Billis	1986	Brazil	NA	Hospital	180	Range, 40–88	45	25.0	3–5	[27]
Yatani	1988	Japan	1965–1979	Hospital	576	Mean, 67.6	NA	22.5	3	[39]
Yatani	1988	Japan	1982–1986	Hospital	660	Mean, 68.7	NA	34.6	3	[39]
Takahashi	1992	Japan	NA	Hospital	29	≥90	17	58.6	3–4	[40]
Gu	1994	China	1989–1992	Hospital	381 (including 60 RCP)	NA	21	5.5	5	[41]
Zare–Mirzaie	2012	Iran	2008–2009	Hospital	149	Mean, 64.5	14	9.4	4	[42]
Zlotta	2013	Japan	2008–2011	Hospital	100	Mean, 68.5	35	35.0	4	[8]
Kimura	2016	Japan	1983–1987	Hospital	501	Mean, 63.5	104	20.8	5	[7]
Inaba	2020	Japan	2009–2017	Hospital	182	Median, 72	71	39.0	5	[43]

## Data Availability

No new data were created or analyzed in this study. Data sharing is not applicable to this study.
